# An electrophysiological validation of stochastic DCM for fMRI

**DOI:** 10.3389/fncom.2012.00103

**Published:** 2013-01-18

**Authors:** J. Daunizeau, L. Lemieux, A. E. Vaudano, K. J. Friston, K. E. Stephan

**Affiliations:** ^1^Motivation, Brain and Behaviour Group, Brain and Spine InstituteParis, France; ^2^Wellcome Trust Centre for Neuroimaging, University College LondonLondon, UK; ^3^Institute of Neurology, University College LondonLondon, UK; ^4^Department of Biomedical science, Metabolism, and Neuroscience, University of Modena and Reggio Emilia, NOCSE HospitalModena, Italy; ^5^Translational Neuromodeling Unit, Institute for Biomedical Engineering, University of Zurich and ETH ZurichZurich, Switzerland; ^6^Laboratory for Social and Neural Systems Research, Department of Economics, University of ZurichZurich, Switzerland

**Keywords:** dynamic causal modeling, neural noise, EEG, fMRI, effective connectivity, neural field, separation of time scales

## Abstract

In this note, we assess the predictive validity of stochastic dynamic causal modeling (sDCM) of functional magnetic resonance imaging (fMRI) data, in terms of its ability to explain changes in the frequency spectrum of concurrently acquired electroencephalography (EEG) signal. We first revisit the heuristic model proposed in Kilner et al. (2005), which suggests that fMRI activation is associated with a frequency modulation of the EEG signal (rather than an amplitude modulation within frequency bands). We propose a quantitative derivation of the underlying idea, based upon a neural field formulation of cortical activity. In brief, dense lateral connections induce a separation of time scales, whereby fast (and high spatial frequency) modes are enslaved by slow (low spatial frequency) modes. This slaving effect is such that the frequency spectrum of fast modes (which dominate EEG signals) is controlled by the amplitude of slow modes (which dominate fMRI signals). We then use conjoint empirical EEG-fMRI data—acquired in epilepsy patients—to demonstrate the electrophysiological underpinning of neural fluctuations inferred from sDCM for fMRI.

## Introduction

In addition to localizing brain regions that encode specific sensory, motor, or cognitive processes, contemporary neuroimaging research tries to understand how information is transmitted within brain networks (Sporns, 2010). Ultimately, the ambition is to understand the nature of the information that neuronal populations pass on to each other. This speaks to the notion of *functional integration*, which views cognitive function as resulting from information exchange within brain networks (Tononi et al., 1994). This means one has to understand the directed influence or *effective connectivity* that brain systems (e.g., cortical areas, neuronal populations, or single neurons) exert on each other. Analyzing effective connectivity rest on models that formalize assumptions about how neuronal systems are wired and how they respond to different stimuli. These models are then used to interpret brain responses measured using, e.g., functional magnetic resonance imaging (fMRI) or magneto-/electroencephalography (M/EEG). We refer to Valdes-Sosa et al. (2011) for a comprehensive review on effective connectivity, and its relationship with influence and causality.

In the context of M/EEG, detailed biophysical knowledge about the activity of neural ensembles has led the community to develop and validate dynamical models that can describe macro-scale dynamics in great detail (cf. Deco et al., 2008; Coombes, 2010; Bressloff, 2012). Most of these models are inspired by approaches in statistical physics based on the notion of a *mean field*, i.e., the idea that interactions among ensembles of neurons can be captured by summary statistics (i.e., moments of the relevant distribution). Dynamic causal modeling [DCM, see Daunizeau et al. (2011) for a recent review] for M/EEG embeds these models into a formal (Bayesian) statistical framework that allows for parameter estimation and model comparison when analyzing evoked or induced responses. In this context, the approach has proven successful in exploiting the realism of these biophysical models to capture experimental effects of cognitive manipulations in terms of network plasticity (e.g., Garrido et al., 2007 or Moran et al., 2011).

Even more established is the use of DCM for fMRI data, for which the approach was originally proposed by Friston et al. (2003), building on previous advances in the biophysics of hemodynamic processes (Buxton et al., 1998). Interestingly, fMRI paradigms elicit neural responses that unfold on a much slower time scale (seconds) than that typical of electrophysiological measurements (milliseconds). In contrast to hemodynamic processes, relatively little is known about the biophysical underpinning of these neuronal responses, which has precluded the use of detailed (realistic) models of neural dynamics in DCM for fMRI. Instead, the slow (and widespread) dynamics of interacting brain regions are captured by phenomenological models that are much simpler than those employed for M/EEG data. Even though these models serve as a reference when evaluating fMRI effective connectivity methods (Smith et al., 2011), their simplicity may impose limitations on the ensuing inferences (but see, for example, David et al., 2008 or Brodersen et al., 2011). This is why we have previously proposed variational Bayesian approaches that can deal with some of the (unavoidably simplifying) model assumptions by introducing random fluctuations of physiological states (Friston et al., 2008, 2010; Daunizeau et al., 2009b). Initial applications of this “stochastic” version of DCM [stochastic dynamic causal modeling (sDCM)] have established some face validity in the context of fMRI data analysis. For example, Friston et al. (2011) showed how sDCM can be used in combination with *post-hoc* model comparison (Friston and Penny, 2011) to explore large model spaces, and Li et al. (2011) examined the smoothness of the underlying neural fluctuations. In Daunizeau et al. (2012), we provide a comprehensive comparison of deterministic and stochastic DCM, in terms of parameter estimation and model comparison. In particular, we showed how accounting for random effects on the system's dynamics can improve network identification by exploiting the decorrelation of neural time series induced by neural noise.

However, sDCM for fMRI has its own methodological challenges and still lacks solid experimental validation. On the one hand, the ability of sDCM for fMRI to discriminate between the respective contributions of neuronal noise and measurement noise in the observed fMRI signal depends on modeling assumptions (Roebroeck et al., 2011; Daunizeau et al., 2012). On the other hand, it is yet unclear how to formally relate the neural states of DCM for fMRI to electrophysiological measures of activity. In this note, we use EEG data to provide empirical evidence for the predictive validity of sDCM for fMRI data. We therefore place this work in the context of the ongoing debate regarding the hemodynamic correlate of the EEG [see Rosa et al. (2010a) for a recent critical review of this literature]. Here, we appeal to neural field theory (Wilson and Cowan, 1972, 1973; Lopes da Silva et al., 1973; Nunez, 1974; Amari, 1975, 1977; Freeman, 1975; Jirsa and Haken, 1996) to revisit the heuristic hemodynamic correlate of EEG signals originally proposed in Kilner et al. (2005). In essence, this hypothesis suggests that the fMRI signal is correlated with the frequency modulation of the EEG (where larger fMRI signals reflect a shift of the EEG spectrum toward higher frequencies). This is based on the idea that slow dynamics underpinning fMRI responses reflect the instantaneous frequency of oscillating modes of activity (Cabral et al., 2011; Friston et al., 2011). Here, we show how slow macroscopic modes of activity that emerge from intrinsic (within region) connections may modulate the frequency spectrum of fast dynamical modes that contribute to the EEG signal. Guided by this notion, we obtain estimates of neural dynamics from fMRI data using sDCM and ask whether these estimates predict the spectral behavior of concurrent EEG data—in terms of the temporal changes in its center frequency (above and beyond physiological confounds). This enables us to establish the predictive validity of sDCM for fMRI data, in relation to electrophysiological responses.

This paper comprises three sections. In the first, we present the neural field model that motivates a separation of time scales and the ensuing heuristic hemodynamic correlate of the EEG. The second section is an empirical demonstration that serves to evaluate the predictive validity of stochastic DCM for fMRI for the ensuing electrophysiological correlates. We close with a discussion of the implications of our findings in the final section.

## Model: revisiting the heuristic hemodynamic correlate of EEG

In this section, we first introduce a neural field model of a cortical region, and show how its spatiotemporal pattern of activity can be decomposed into canonical dynamic modes that have distinct time scales. In brief, slow macroscopic neural states control the rate of change of fast components. This motivates both the separation of time scales that underlies the generative model of sDCM for fMRI (Friston et al., 2011) and the heuristic hemodynamic correlate of EEG (Kilner et al., 2005). In brief, this section serves as a partial theoretical derivation of sDCM for fMRI, from the perspective of biologically-informed (neural field) models of macroscopic electrophysiological activity.

### Neural fields and separation of time scales

We start with a description of the dynamics of single neurons within an ensemble, which we describe in terms of the probability density function *p*(ς) of neuronal post-synaptic membrane potentials (PSP) ς. We assume that neurons fire an action potential if their PSP surpasses a depolarization threshold χ. This means that 〈*H*(ς−χ)〉 represents the average (mean field) firing rate over the ensemble, where *H* is the Heaviside step function and 〈 〉 is the expectation under the ensemble density. Using the central limit theorem, one can show that the average firing rate over the ensemble is determined mainly by the first two moments of the PSP ensemble density *p*(ς) (Liley et al., 2002; Marreiros et al., 2008):

(1) $$\begin{array}{l} \langle H(\varsigma -\chi )\rangle =\int_{\chi }^{\infty }p(\varsigma )d\varsigma \approx S(\eta )=\frac{1}{1+\exp(-\rho (\eta -\chi ))}\\ \;\eta =\langle \varsigma \rangle \\ \;\rho^{-2}=\frac{3}{\pi^{2}}\langle {\left(\varsigma -\eta \right)}^{2}\rangle \end{array}$$

where *S* is a sigmoid function and ρ is proportional to the inverse of the standard deviation (dispersion) of depolarization within the ensemble. This is a statistical (mesoscopic) summary of neuronal ensemble activity, corresponding roughly to a cortical macrocolumn (about 100,000 neurons and 1 mm^2^ of cortex). For our modeling purposes, this summary statistics is now taken to the macroscale, where each ensemble or column has a position **r** on the 2D-cortical manifold. At this macroscopic scale, states like depolarization can be regarded as a continuum or *field* (Jirsa and Haken, 1996; Liley et al., 2002; Coombes et al., 2007; Daunizeau et al., 2009a; Pinotsis et al., 2012), which is a function of space and time: η(*t*) → η(**r**, *t*). This means we can describe the local average input and output activity of neurons using the depolarization field η(**r**, *t*) and the sigmoid function. The spatiotemporal dynamics of the neural field essentially depends on how local ensembles influence each other—as described below.

**Figure 1:**
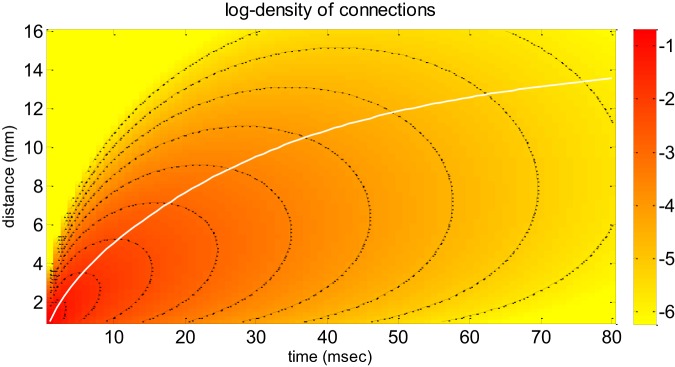
**Neural field model: the dispersive propagator.** This figure depicts the dispersive propagator of Equation (4) as a function of both time (x-axis) and distance (y-axis), in terms of the density 2π*rG*(*r, t*) of synaptic connections that are reached by an action potential emitted at *r* = *t* = 0 (on a logarithmic scale). Note that the 2π*r* scaling arises because of radial symmetry in 2D (Bojak and Liley, 2010). The white line shows the average distance, at which activity is propagated as a function of time. The black lines are contour lines of the connection density. These can be used to eyeball how fast the density attenuates (e.g., along the average distance path).

We now consider a region of the cortical manifold that is defined by its continuous domain *D*, over which the neural field η(**r**, *t*) is defined. The field is considered to result from the temporal convolution of pre-synaptic input μ(**r**, *t*) as follows:

(2) $$\begin{array}{l} \eta (r,t)=\int_{-\infty }^{\infty }J(t-t^{\prime })\mu (r,t)dt^{\prime }\\ \;=J⊗\mu (r,t)\\ \;J(t)=\{\begin{array}{cc} \gamma \frac{t}{\tau }\exp\left(-\frac{t}{\tau }\right) & t\geq 0\\ 0 & t<0 \end{array}, \end{array}$$

where *J* is the synaptic impulse response function, τ is a time-constant, and γ controls the maximum PSP following an action potential. The maximum PSP and time-constant summarize the microscopic properties of the ensemble (Jansen and Rit, 1995). Because action potentials propagate with a finite conduction velocity, the mean rate of arrival of pre-synaptic impulses can be expressed as a time-delayed integral (spatiotemporal convolution) of local firing rates:

(3) $$\begin{array}{l} \mu (r,t)=\int_{-\infty }^{\infty }\int_{D}G(|r-r^{\prime }|,t-t^{\prime })S(\eta (r^{\prime },t^{\prime }))dr^{\prime }dt^{\prime }\\ \;=G⊗S{}^{\circ}\eta (r,t), \end{array}$$

where *D* is the domain, over which the neural field is defined, and *G* is a homogeneous Green function that acts as a spatiotemporal convolution operator. Let *r* = |**r**−**r**'| be the distance between two points with positions **r** and **r**'. Below, we follow Bojak and Liley (2010) and parameterize the Green function as follows:

(4) $$G(r,t)=\frac{G^{0}}{4\pi }\frac{1}{\sigma^{2}}t^{-1}\exp\left(-\frac{r^{2}+{\bar{\nu }}^{2}t^{2}}{2\sigma \bar{\nu }t}\right)H(t),$$

where the Heaviside step function endows Equation (3) with temporal causality and *G*^0^ is the total number of synaptic connections. This *dispersive propagator* assumes a Gaussian falloff in the density of synaptic connections and accounts for a certain variability in velocities ($\bar{\nu }$ is the mode of the velocity distribution at “large distance,” i.e., when *r*/σ → ∞). The spatial scale of lateral connectivity is controlled by the spatial dispersion σ. Figure 1 depicts the dependence of the dispersive propagator with respect to space and time (see also Liley et al., 2012—Figure 3).

In addition to its plausible form, this homogeneous Green function has proven very useful, in that it has an analytical Fourier transform. Applying the Fourier transform back and forth to Equations (2) and (3) yields the following partial differential equation (see Bojak and Liley, 2010):

(5) $$\begin{array}{l} \left(\frac{\bar{\nu }}{2\sigma }+\frac{\partial }{\partial t}-\frac{\bar{\nu }\sigma }{2}\nabla^{2}\right)\mu (r,t)=\frac{G^{0}\bar{\nu }}{2\sigma }S(\eta (r,t))\\ \left(\tau^{2}\frac{\partial^{2}}{\partial t^{2}}+2\tau \frac{\partial }{\partial t}+1\right)\eta (r,t)=\tau \gamma \mu (r,t). \end{array}$$

The first partial differential equation describes the diffusion of action potentials of spikes (through the Laplacian operator) and derives from the lateral connectivity kernel in the Green function. The second ordinary differential equation derives from Equation (2) and describes synaptic transmission; i.e., how propagated pre-synaptic firing is accumulated locally to produce depolarization.

The solution to Equation (5) can be decomposed into canonical dynamical modes, by projecting the neural field on the set of eigenvectors *w*^(*k*)^(**r**) of the Laplacian operator defined over the domain *D* (see Daunizeau et al., 2009a). These spatial modes solve the Laplacian eigenvalue problem, i.e.,: ∇^2^ *w*^(*k*)^ (**r**) = λ_*k*_ *w*^(*k*)^ (**r**), where the distribution of eigenvalues λ is such that: λ_*k*_ <λ_0_ = 0. They form a complete and orthonormal basis function set for the neural field, allowing for an eigen-decomposition of the field, as follows:

(6) $$\begin{array}{l} \eta (r,t)=\sum_{k}z_{1}^{\left(k\right)}(t)w^{\left(k\right)}(r)\\ \mu (r,t)=\sum_{k}z_{2}^{\left(k\right)}(t)w^{\left(k\right)}(r), \end{array}$$

here, the dynamics of the spatial modes are defined as:

(7) $$\begin{array}{l} z_{1}^{\left(k\right)}(t)=\int_{D}w^{\left(k\right)}(r)\eta (r,t)dr\\ z_{2}^{\left(k\right)}(t)=\int_{D}w^{\left(k\right)}(r)\mu (r,t)dr. \end{array}$$

Furthermore, it can be shown that the dynamics of the spatial modes (Equation 5) can be approximated as third-order ordinary differential equations that are coupled through the fundamental mode *k* = 0 (see Appendix 1):

(8) $$\begin{array}{l} \;{\dot{z}}^{\left(k\right)}=\left[\begin{array}{c} {\dot{z}}_{1}^{\left(k\right)}\\ {\dot{z}}_{2}^{\left(k\right)}\\ {\dot{z}}_{3}^{\left(k\right)} \end{array}\right]\\ \;=\left[\begin{array}{ccc} 0 & 0 & 1\\ c\left(z_{1}^{\left(0\right)}\right) & \frac{\bar{\nu }}{2\sigma }\left(\sigma^{2}\lambda_{k}-1\right) & 0\\ -\frac{1}{\tau^{2}} & \frac{\gamma }{\tau } & -\frac{2}{\tau } \end{array}\right]z^{\left(k\right)}+O\left(z^{2}\right)\\ c\left(z_{1}^{\left(0\right)}\right)\equiv \frac{\rho G^{0}\bar{\nu }}{2\sigma }S\left(z_{1}^{\left(0\right)}\right)\left(1-S\left(z_{1}^{\left(0\right)}\right)\right) \end{array}$$

here, *z*_3_ ≡ ż_1_, and *cz*^(0)^_1_) is a non-linear function of the fundamental mode *z*^(0)^_1_ that mediates the coupling between modes describing firing rate and depolarization. Roughly speaking, each mode decays at a rate that is proportional to $\sigma \bar{\nu }\lambda_{k}$. As a consequence, high propagation velocities in large cortical regions dissipate the spatial modes quickly, with the exception of the fundamental mode, which has a zero eigenvalue. More generally, smooth eigenmodes (low spatial scales) will be associated with slow dynamics (see, e.g., Schultze-Kraft et al., 2011). Note that the natural time scale of the fundamental mode can be very slow, if the conduction velocity $\bar{\nu }$ becomes small compared to the dispersion of lateral connections σ (i.e., if $\bar{\nu }/\sigma \to 0$). In this case, the fundamental mode *z*^(0)^ will decay so slowly that it will predominate over other stable (higher spatial frequency) modes *z*^(*k*)^, which disappear as soon as they are created. This means that Equation (8) effectively instantiates a separation of time scales, where the slowest mode “enslaves” the fast modes (Haken, 1983) through the coupling function *cz*^(0)^_1_). This slaving effect is such that the frequency profile of fast modes (*k*>0) is controlled by the amplitude of slow modes (*k* = 0).

### Modeling local activation in terms of EEG frequency modulation

Equation (8) expresses the Jacobian of eigenmode dynamics as a function of the fundamental mode of activity *z*^(0)^_1_ within a given brain region, over which the neural field is defined. This means that slow fluctuations in the fundamental mode *z*^(0)^_1_ should be associated with changes in the EEG frequency spectrum P(ω), which derives from the Laplace transform of Equation (8) (see Appendix 2):

(9) $$\text{P}(\omega )\propto {\left|\sum_{k\;\leq \;k_{c}}\frac{\begin{array}{l} \left(i\omega +\frac{\bar{\nu }}{2\sigma }\left(1-\sigma^{2}\lambda_{k}\right)\right)\\ \left(i\omega +\frac{2}{\tau }+1\right)+\frac{\gamma }{\tau } \end{array}}{\begin{array}{l} \left(i\omega +\frac{\bar{\nu }}{2\sigma }\left(1-\sigma^{2}\lambda_{k}\right)\right)\\ \left(i\omega \left(i\omega +\frac{2}{\tau }\right)+\frac{1}{\tau^{2}}\right)-\frac{\gamma }{\tau }c\left(z_{1}^{\left(0\right)}\right) \end{array}}\right|}^{2}$$

where *k*_*c*_ is the cut-off order induced by the skull's low electrical conductivity. Here, the summation is over the fast modes (up to *k*_*c*_ = 16), and we have assumed that EEG responses reflect postsynaptic potentials, i.e., *z*^(*k*)^_1_. Similarly to Kilner et al. (2005), let us now define the EEG center frequency $\bar{\omega }$ as the first moment of the (normalized) frequency power spectrum $\bar{\text{P}}(\omega )$:

(10) $$\begin{array}{l} \bar{\text{P}}(\omega )=\frac{\text{P}(\omega )}{\int_{\omega_{0}}^{\infty }\text{P}(\omega )d\omega }\\ \;\bar{\omega }\equiv \int_{\omega_{0}}^{\infty }\omega \bar{\text{P}}(\omega )d\omega \end{array}$$

By construction, normalized frequency power $\bar{\text{P}}(\omega )$ is the proportion of total power attributed to any given frequency of interest. Thus, one can think of it in terms of a probability density function over frequencies, whose first-order moment would be the center frequency. Intuitively, an increase in the center frequency $\bar{\omega }$ signals a shift of frequency power toward higher frequencies. Note that the center frequency is not the main peak in the frequency spectrum. Instead, variations of $\bar{\omega }$ over time quantify frequency modulations of the EEG signal. The key idea here is that the EEG center frequency $\bar{\omega }$ is a function of the slow eigenmode dynamics *z*^(0)^_1_, and its modulation over time thus follows the slow eigenmode dynamics *z*^(0)^_1_, i.e., $\bar{\omega }=\bar{\omega }\left(z_{1}^{\left(0\right)}\right)$.

**Figure 2:**
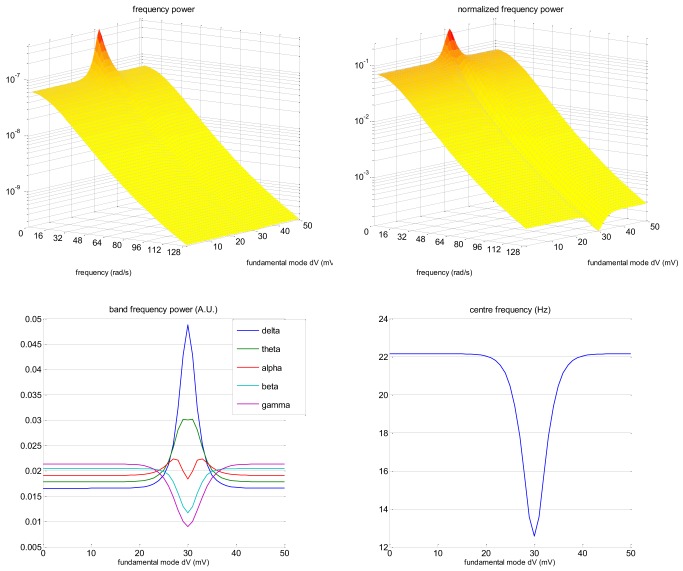
**Neural field model: EEG frequency modulation**. This figure shows the effect of changes in the mean membrane depolarization fundamental eigenmode *z*^(0)^_1_ on the frequency content of the EEG signal. **Upper-left:** EEG frequency spectrum P(ω) (z-axis) as a function of frequency ω (x-axis) and eigenmode amplitude *z*^(0)^_1_ (y-axis). **Upper-right:** Normalized frequency spectrum $\bar{\text{P}}(\omega )$ (cf. Equation 10, same format). **Lower-left:** Magnitude of standard EEG frequency bands (y-axis; blue: delta, green: theta, red: alpha, magenta: beta, violet: gamma) as a function of the eigenmode amplitude *z*^(0)^_1_ (x-axis). **Lower-right:** Centre frequency $\bar{\omega }$ (y-axis) as a function of the eigenmode amplitude *z*^(0)^_1_ (x-axis). Note that here, the action potential firing threshold χ is 30 mV.

Figure 2 depicts the frequency modulation of the EEG signal induced by changes in the slow eigenmode *z*^(0)^_1_. The frequency power spectrum P (ω) given in Equation (9) was evaluated using the parameter values given in Table 1 below. One can see that when the slow eigenmode tends toward the action-potential firing threshold (*z*^(0)^_1_ → χ), there is both a power increase in the low frequencies and a decrease in the high frequencies (cf. increase in the delta/theta/alpha band, and decrease in the beta/gamma band). This shift toward lower frequencies can be seen most clearly on the normalized frequency power $\bar{\text{P}}(\omega )$. As a consequence, the center frequency $\bar{\omega }$ increases as the slow eigenmode gets further away from the action potential firing threshold. In our example, this induces a change of about 10 Hz in the EEG center frequency. Note that the precise numerical value of the center frequency $\bar{\omega }$ (as well as its susceptibility to the eigenmode *z*^(0)^_1_) depends upon the neural field's parameters (cf. Table 1). In fact, using more realistic neural fields models (accounting for, e.g., excitatory and inhibitory interneurons, see Pinotsis et al., 2012) would profoundly change the shape of the frequency power spectrum. However, the qualitative effect of neural activation upon the center frequency is qualitatively invariant to such changes. We will comment on this in the “Discussion” section.

**Table 1:** **Neural field parameters**.

**Constant**	**Physical meaning**	**Value**
γ	Maximum post-synaptic impulse response	8 mV
τ	Post-synaptic impulse response decay rate	4 ms
*G*^0^	Total number of synaptic connections	2000
σ	Spatial decay rate of synaptic connections	3 mm
ρ	Inverse of the within-ensemble PSP standard deviation	0.54 mV^-1^
χ	Action potential firing threshold (w.r.t. PSP steady-state)	30 mV
$\bar{\nu }$	Average conduction velocity	3 m/s

*These parameter values were taken from Pinotsis et al. (2012)*.

### The link between EEG time-frequency response and fMRI temporal response

In this paper, we assume that the slow macroscopic dynamics *x*^(*n*)^ underlying fMRI data in a given region of interest follow the fundamental eigenmode *z*^(0)^_1_ of its membrane depolarization cortical field. The motivations for this assumption include the following:

- Its relative simplicity.
- Sensitivity to input versus output spiking activity: there is a consensus now on the fact that mean membrane depolarization (as opposed to e.g., firing rate) drives hemodynamic changes [see Logothetis and Wandell (2004) for a review on the relationship between local field potentials and the BOLD signal).
- Energy budget: both the slow time scale and the low spatial frequency of the fundamental eigenmode make it likely to be the main driver of induced BOLD changes, when compared with faster (and higher spatial frequency) eigenmodes. This is because blood flow increases in local vascular trees are induced by coherent spatio-temporal summation of neural activity (see e.g., Daunizeau et al., 2010; Rosa et al., 2010a,b; Schmuel, 2010).
- It relates to theoretical accounts on critical slowing of high-dimensional dynamical systems, which has recently been used to motivate the rate of change of neural states in stochastic DCM for fMRI (Friston et al., 2010).

We will further comment on this point in the “Discussion” section.

In the previous section, we have described how a separation of time scales could arise from local interactions at the level of a cortical region. It turns out that this result can be generalized to a set of coupled local neural fields, in that the fundamental mode mediates the extrinsic (between-region) coupling. In other words, the influence different neural fields exert on each other expresses itself through the coupling of their respective fundamental modes, which locally drive higher order modes (as in Equation 8). Deriving the precise dynamical properties of coupled neural fields is beyond the scope of the present study. However, one can invoke the slaving effect when considering coupled (distal) brain regions, the network properties of which shape the temporal dynamics of local fundamental eigenmodes *z*^(0)^_1_. This means that long-range connections control the frequency profile of brain regions responses (see e.g., Bojak et al., 2011), through their effect on their respective fundamental eigenmodes *z*^(0)^_1_. Since we did not consider coupled neural fields in the above treatment, we replace their (unknown) evolution function with a first-order Taylor expansion on slow neural states, yielding the following Langevin equation for stochastic DCM (see Daunizeau et al., 2012):

(11) $${\dot{x}}^{\left(n\right)}\approx Ax^{\left(n\right)}+v$$

where **θ**^(*n*)^ = {**A**_*ij*_} are neuronal parameters that measure extrinsic coupling strengths. Here, we have neglected non-linear interaction terms that act as gating factors on the network connections *x*^(*n*)^ (cf. Stephan et al., 2008). The influence of the fast modes on the motion of slow modes **x**^(*n*)^ is expressed via fluctuations **v**, which are comparatively much faster. This allows us, in the context of sDCM for fMRI, to treat **v** as stochastic (neural) noise and place priors *p*(**v**|*m*) on its generalized motion (see below).

In contradistinction, typical evoked EEG responses are driven by a mixture of slow and fast modes. Note that the relative contribution of the slow and fast modes is imbalanced (toward fast modes), simply because the summation span all eigenmodes up to cut-off order *k*_*c*_. In fact, typical evoked EEG responses disappear within a second or so of peristimulus time; i.e., their limit frequency has an order of magnitude similar to the fMRI sampling rate (about 1 Hz).

Taken together, we expect to see concurrent changes in the slow neural states *x*^(*n*)^ that drive BOLD and in the EEG power spectrum P (ω). However, from the above section, we know that the underlying relationship is non-linear and depends upon intrinsic properties of local neural fields, such as the size of active brain regions and the spatial decay rate of synaptic connections (cf. Equation 9). In addition, the respective contribution of each region to the scalp EEG power spectrum is virtually unknown, as this would require solving the so-called EEG inverse problem (see Rosa et al., 2010a,b). These issues conspire with the unavoidable simplifications of our model to make the prediction of the full EEG frequency spectrum practically irrelevant. In the following, we will thus focus on the EEG center frequency $\bar{\omega }$, which captures global changes in the frequency spectrum. For the sake of simplicity, in the remainder of this work, we will invoke a first-order Taylor expansion of the following form:

(12) $$\bar{\omega }\left(x^{\left(n\right)}\right)=\bar{\omega }(0)+{\frac{\partial \bar{\omega }}{\partial x^{\left(n\right)}}|}_{0}x^{\left(n\right)}+O\left(x^{2}\right)$$

where the gradient ${\partial \bar{\omega }/\partial x^{\left(n\right)}|}_{0}$ measures the susceptibility of the center frequency to the slow neural states. This quantity will be directly estimated from experimental data (along with the intercept $\bar{\omega }(0)$, which acts as a confound). Testing for Equation (12) effectively bypasses the dependency of the EEG power spectrum to the respective contribution each local neural field included in the network, as well as their biophysical properties, and only retains the qualitative prediction of the model.

#### Summary

In this section, we have used the neural field formulation of local neural activity to demonstrate an emergent separation of temporal scales, in which the slow fluctuations of modes or patterns of depolarization enslave faster modes. Crucially, the amplitude of slow modes (which we associate with fMRI signals) and modulate the frequencies of fast modes (which we associate with EEG signals). This dynamical behavior is based upon a plausible mean field approximation to macroscopic neuronal activity on the cortical surface and, within the setting, provides a formal verification of the heuristic in Kilner et al. (2005), linking fMRI signals to changes in the center frequency of electrophysiological signals. In the next section, we will use stochastic DCM to obtain estimates of macroscopic (slow) neural fluctuations from fMRI data. We then test whether these estimates can predict fluctuations in concurrently measured EEG data—in terms of its center frequency, as suggested by the neural field treatment above (Equations 9–12).

## Empirical data: resting state and generalized spike and wave (GSW) activity

In this section, we conduct an sDCM analysis of data from patients with epilepsy who exhibited generalized spike and wave (GSW) activity, while undergoing concurrent EEG/fMRI measurements in the scanner. We first describe the EEG/fMRI acquisition and conventional activation analysis. We then describe the sDCM analysis of fMRI data and ensuing validation using EEG data.

### EEG/fMRI acquisition and activation analysis

These data were part of a previous neuroimaging study of idiopathic generalized epilepsy (Hamandi et al., 2006; Vaudano et al., 2009), whose acquisition protocol and data pre-processing are briefly summarized here.

Ten-channel EEG (10–20 system) was recorded using MR-compatible equipment, along with bipolar electrocardiogram. After filtering and artifact correction (Krakow et al., 2000), the onsets and offsets of GSW activity were identified by two experts (see Vaudano et al., 2009 for details). Seven hundred T2^*^-weighted single-shot gradient echo echo-planar images (TE = 40 ms, TR = 3 s, 21 interleaved axial slices of 5 mm thickness, FOV = 24 × 24 cm^2^, 64 × 64 matrix) were acquired over a 35 min session with a 1.5 Tesla MRI scanner (Horizon EchoSpeed, General Electric). Patients were asked to rest with their eyes closed and to keep still. FMRI data were pre-processed using SPM8 (http://www.fil.ion.ucl.ac.uk/spm/). EPI time series were realigned and spatially smoothed with an 8 mm FWHM isotropic Gaussian kernel and spatially normalized to the standard anatomical space. For each patient, a general linear model (GLM) was constructed to test for the presence of regional GSW-related BOLD changes. Periods of GSW activity were modeled as blocks, beginning at GSW onset and terminating at their offset. The GSW regressors were then convolved with the canonical hemodynamic response function (plus temporal and dispersion derivatives) before inclusion in the GLM. In addition, we included slow drifts (Fourier basis function set), as well as motion-related effects (head and eye movements), cardiac-related effects (see Liston et al., 2006) and scan-nulling regressors (modeling inter-scan motion events larger than 0.2 mm, cf. Lemieux et al., 2007) as confounding factors.

On average, the GLM design matrices contained about 100 confounds regressors. Significant positive and negative GSW-related BOLD responses were identified by means of an *F*-contrast on the GSW regressors for the nine patients included in this study. SPMs were thresholded at *p* < 0.05 (FWE whole-brain corrected) to define three regions of interest (ROIs), which were implicated in the initiation and termination of GSW discharges in all patients: thalamus, prefrontal cortex (PFC), and precuneus. A summary time series was derived for each ROI by computing the first eigenvariate of all suprathreshold voxel time series within a 10 mm of the ROI centers. The time series were adjusted for all confounding effects included in the GLM analysis.

### Validating the sDCM macroscopic neural states estimates with EEG: methods

In addition to the neural evolution function given in Equation (11), DCM for fMRI requires the specification of an additional set of hemodynamic states that couple neural dynamics to observed BOLD signal changes:

(13) $${\dot{x}}^{\left(h\right)}=\left[\begin{array}{c} x^{\left(n\right)}-\kappa_{s}x_{1}^{\left(h\right)}-\kappa_{f}\left(e^{x_{2}^{\left(h\right)}}-1\right)\\ x_{1}^{\left(h\right)}e^{-x_{2}^{\left(h\right)}}\\ \frac{1}{\tau_{0}}\left(e^{x_{2}^{\left(h\right)}}-e^{x_{3}^{\left(h\right)}/\alpha }\right)e^{-x_{3}^{\left(h\right)}}\\ \frac{1}{\tau_{0}}\left(\frac{e^{x_{2}^{\left(h\right)}-x_{4}^{\left(h\right)}}\left(1-{\left(1-E_{0}\right)}^{e^{-x_{2}^{\left(h\right)}}}\right)}{E_{0}}-e^{\left(1-\alpha \right)x_{3}^{\left(h\right)}/\alpha }\right) \end{array}\right]$$

here, *x*^(*n*)^ represents regional neural activity, whose dynamics is given in Equation (11). Equation (13) expresses changes in hemodynamic states **x**^(*h*)^, as a response to a neural perturbation *x*^(*n*)^ and as a function of hemodynamic parameters **θ**^(*h*)^ = {κ_*s*_, κ_*f*_, α, τ_0_, *E*_0_, ε_0_} (for details, see the appendix in Stephan et al., 2008). Finally, one has to specify the observation mapping from hemodynamic states **x**^(*h*)^ to observed local BOLD changes *y* (Stephan et al., 2007):

(14) $$y=V_{0}(4.3\nu_{0}E_{0}TE\left(1-e^{x_{4}^{\left(h\right)}}\right)+\varepsilon_{0}r_{0}E_{0}TE\left(1-e^{x_{4}^{\left(h\right)}-x_{3}^{\left(h\right)}}\right)+\left(1-\varepsilon_{0}\right)\left(1-e^{x_{3}^{\left(h\right)}}\right))+\varepsilon ,$$

where ε is an additive measurement noise, and the parameters are defined in Table 2 below.

**Table 2:** **Prior means of hemodynamic parameters and acquisition-related constants for fMRI at 1.5 T**.

Constant	Physical meaning	Value
κ_*s*_	Vasodilatory signal decay rate	0.65 Hz
κ_*f*_	Vasodilatory signal feedback rate	0.41 Hz
τ_0_	Mean transit time	2 s
α	Vessel stiffness	0.32
*E*_0_	Oxygen extraction fraction at rest	0.34
*V*_0_	Venous volume fraction at rest	4
ν_0_	Frequency offset	40.3 Hz
*TE*	EPI echo time	0.04 s
*r*_0_	Intravascular relaxation rate	25 Hz
ε_0_	Ratio of intra- and extra-vascular signals	1

*Note that θ^(h)^ = {κ_s_, κ_f_, α, τ_0_, *E*_0_, ε_0_} are estimated from the data (i.e., have a positive prior variance), whereas the remaining parameters are treated as constants*.

In brief, the predicted data **y** depend non-linearly on the unknown model variables Θ = {**x**, θ}, through Equations (11), (13), and (14). This model (as well as statistical assumptions about measurement noise ε) is encoded in the likelihood function *p*(**y**|Θ,*m*). Priors *p*(Θ|*m*) specify our assumptions about the magnitude of state noise, evolution parameters and observation parameters—where the prior means of the hemodynamic parameters of Equations (13) and (14) are given in Table 2:

Inverting the generative model *m* means (1) approximating the conditional density *p*(Θ|**y**, *m*) of unknown variables Θ given the set of sampled measurements **y** and (2) quantifying the model evidence *p*(**y**|*m*). Non-linearities in the generative model eschew exact analytical solutions to this problem, which is finessed using variational approaches that rest on optimizing a free-energy lower bound *F*(*q*) to the model evidence, with respect to an approximate conditional density *q*(Θ):

(15) $$\begin{array}{l} F(q)={\langle \ln p\left(\Theta |m\right)+\ln p\left(y|\Theta ,m\right)-\ln q\left(\Theta \right)\rangle }_{q}\\ \;=\ln p\left(y|m\right)-D_{\text{KL}}\left(q(\Theta );p(\Theta |y,m)\right), \end{array}$$

where *D*_*KL*_ is the Kullback-Leibler divergence and the expectation 〈 〉_*q*_ is taken under *q*. From Equation (15), maximizing the functional *F*(*q*) with respect to *q* minimizes the Kullback-Leibler divergence between *q*(Θ) and the exact posterior *p*(Θ|**y**, *m*). This decomposition is complete in the sense that if *q*(Θ) = *p*(Θ|**y**, *m*), then *F*(*q*) = ln *p*(**y**|*m*). Typically, the iterative maximization of free energy proceeds under the Laplace approximation, where the approximate posterior *q*(Θ)≈ *p*(Θ|**y**, *m*) is assumed to have a Gaussian form (see Friston et al., 2007). We refer to Daunizeau et al. (2009b) for details about the application of the variational Bayesian approach to stochastic DCM.

Following model inversion as described above, inference on hidden states is based on the conditional density *q*(**x**) and thus depends upon the generative model *m*. Usually, in DCM, inference on states or parameters is preceded by model selection. However, our focus was on the validity of sDCM for fMRI with regard to predicting the spectral behavior of concurrently acquired EEG data—based on the idea that slow macroscopic neural states **x**^(*n*)^ control the EEG frequency modulation. We therefore endowed the network with full reciprocal connectivity (no zero entry in matrix **A** of Equation 11) and simply applied the above variational Bayesian approach to derive the first moment $\hat{x}={\langle x^{\left(n\right)}\rangle }_{q}$ of the conditional density *q*(**x**). In the following, $\hat{x}$ thus refers to the sDCM estimate of the slow neural states, given fMRI time series **y** (and under model *m*).

To assess the relationship between EEG frequency modulation and neural states as estimated by sDCM, we constructed a GLM *H*_1_, whose dependent variable was the trajectory $\bar{\omega }$ of the EEG center frequency sampled at each fMRI time sample and whose independent variables were the neural states estimated by sDCM plus confounds (cf. Equation 12):

(16) $$H_{1}:\bar{\omega }=\left[\hat{x}\;X_{0}\right]\left[\begin{array}{c} \beta \\ \beta_{0} \end{array}\right]+e$$

where $\hat{x}$ are the sDCM conditional estimates, **X**_0_ contains the confounds (see below), β and β_0_ are unknown regression coefficients, and **e** are i.i.d. residuals (with zero mean and unknown variance). Parameters β effectively capture the susceptibility of the EEG center frequency with respect to the slow neural states (cf. gradient ${\partial \bar{\omega }/\partial x^{\left(n\right)}|}_{0}$ in Equation 12). They lump together the contributions of the multiple regions that constitute the network as well as biophysical properties of the local neural fields. Parameters β_0_ weight the contribution of the confounding factors to changes in the EEG center frequency. We then tested whether the sDCM neural states predicted EEG frequency modulation above and beyond what could be explained using the confounds. To this end, we used Bayesian model comparison at the group level (Stephan et al., 2009) to quantify the evidence in favor of the full model *H*_1_, relative to a reduced model *H*_0_ that only included the confounds:

(17) $$H_{0}:\bar{\omega }=X_{0}\beta_{0}+e$$

The assessment of sDCM predictive validity thus depends on the definition of the confounds, i.e., uncontrolled sources of variations in the EEG center frequency. In order to investigate the robustness of the statistical relationship between the slow eigenmode dynamics $\hat{x}$ and the EEG center frequency trajectory $\bar{\omega }$, we have defined two different sets of confounds:

- *Slow drifts*: this assumes that the EEG center frequency undergoes slow and unspecific variations due to e.g., skin conductance changes, residual eye blinks artifacts, etc. …. Under this assumption, we set up **X**_0_ similarly to the minimal confounds of a typical fMRI activation analysis, i.e., a Fourier basis function set (16 first harmonics; see Figure 6).
- *Full confounds*: this generalizes the intuition that confounds on the EEG center frequency are similar to those included in the fMRI activation analysis. For example, cardiac and respiratory effects, as well as body movements, could be modeled directly, as potential sources of (nuisance) variations. Under this assumption, we set up **x**_0_ identically to the full confounding matrix used for each subject fMRI activation analysis (cf. section “EEG/fMRI Acquisition and Activation Analysis” above).

In brief, testing for the contribution of sDCM neural estimates $\hat{x}$ on variations of the center frequency $\bar{\omega }$, above and beyond the full confounds is the most conservative of the two alternatives. However, it will be the least sensitive of both models as well; recall that β has only *n*_ROI_ entries, where *n*_ROI_ = 3 is the number of ROIs included in the sDCM analysis (to be compared with the dimension of β_0_). In other words, these two sets of confounds can be motivated on principled grounds. We will thus perform a statistical comparison between *H*_0_ and *H*_1_ that marginalizes over the two sets of confounds.

### Validating neural states estimate obtained by sDCM against EEG

**Figure 3:**
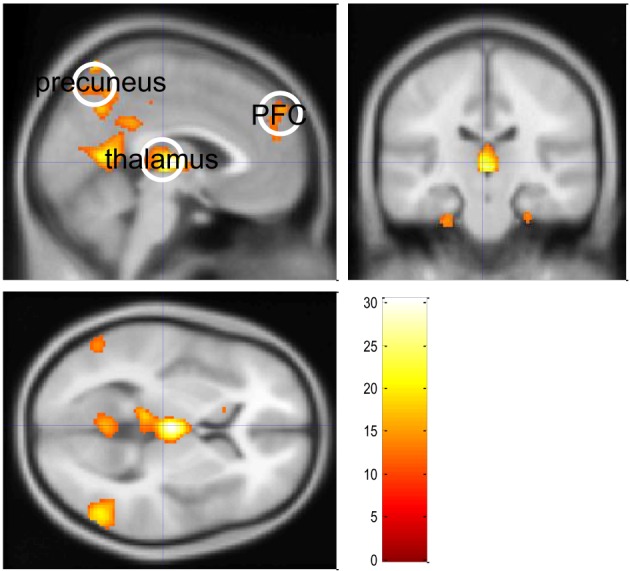
**Absence seizure analysis: regions of interest.** This figure summarizes the standard SPM activation results of a single epileptic patient with (petit mal) absence seizure (the same subject as in Figure 1). Significant (whole brain FWE-corrected, *p* < 0.05) positive and negative GSW-related BOLD responses were identified by means of an *F*-contrast on the GSW regressors. The color bar indicates the range of displayed *F* values.

**Figure 4:**
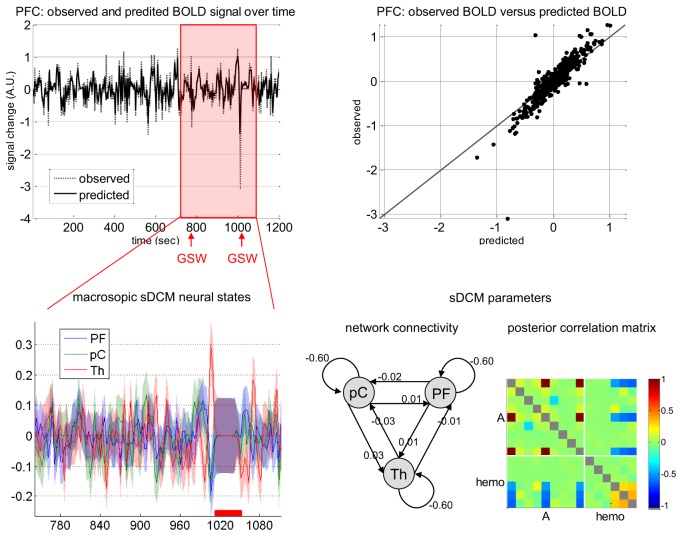
**Absence seizure analysis: sDCM analysis.** This figure summarizes the sDCM analysis of a single subject fMRI data (same patient as in Figure 1). **Upper-left:** Time series of the observed and fitted BOLD signal in prefrontal cortex (PFC) as a function of time. The red shaded area indicates peri-GSW activity. **Upper-right:** Observed (y-axis) versus fitted (x-axis) BOLD signal in PFC. **Lower-left:** Macroscopic sDCM neural states in the three regions of interest (PF, prefrontal cortex; pC, precuneus; Th, thalamus) during peri-GSW activity, as a function of time. The red bar indicates strong head motion, which was modeled using scan-nulling regressors (see main text). **Lower-right:** sDCM conditional density on model parameters (left: first-order moment and ensuing network connectivity, right: posterior correlation matrix). Note: identifiability issues between pairs of parameters manifest as high posterior correlations.

Figure 3 shows the SPM of significant GSW-related BOLD changes (above and beyond physiological confounds), for the first patient of the study (*F*-test, *p* < 0.05, whole-brain corrected). This was used to define the three ROIs included in the sDCM analysis (Figure 4).

One can see that most of the variance of the BOLD response is explained by the stochastic DCM. Focusing on peri-GSW periods, one can see that the conditional density *q*(**x**) shows high temporal variability, with increased uncertainty during head motion. This is expected, as scan-nulling confound regressors effectively prevent sDCM from deriving any information from fMRI data about hidden states during these periods. It is interesting to note that the network connectivity coefficients (elements of the **A** matrix in Equation 11) are small (about 0.02 Hz). This means that the average coupling of these regions (over the entire recording) is rather weak. Importantly, with the (partial) exception of the inhibitory intrinsic self-connections, the posterior correlation matrix (Figure 4) shows that most system parameters are identifiable and not confounded by hemodynamic parameter estimation. Generally, Figure 4 summarizes the motivation for this work—is the temporal variability of sDCM estimates of neuronal activity an artifact of measurement noise, or does it have an electrophysiological underpinning?

**Figure 5:**
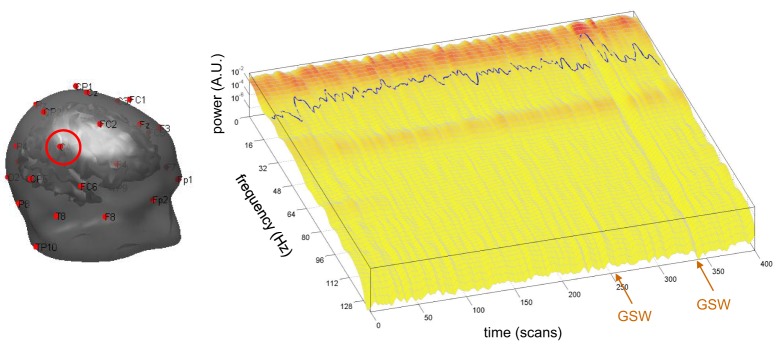
**Absence seizure analysis: derivation of the EEG frequency modulation.** This figure depicts the extraction of the EEG center frequency in a single subject (same patient as in Figure 1). **Left:** The EEG set-up used during the recording session is shown superimposed on the brain and skin surfaces (sensor C4 is highlighted). **Right:** Normalized square root power of windowed Fourier transform (z-axis) of the EEG traces of sensor C4 (x-axis: scanning time, y-axis: instantaneous frequency). The blue line shows the center frequency $\bar{\omega }$ as a function of scanning time *t* (cf. Equation 10 in the main text).

The cross-validation of neural state estimates by sDCM from fMRI data against EEG data requires the extraction of the time-dependent center frequency $\bar{\omega }$ for each channel of each subject, according to Equation (10). This is depicted in Figure 5, for the EEG channel C4 of the same patient in Figures 3, 4.

Recall that comparisons between the experimental and theoretical profiles of the frequency spectrum show differences that prevent meaningful quantitative fitting of Equation (9). For example, the model cannot account for peaks in the alpha band that appear in occipital channels (at least for the neural field parameter values of Table 1; results not shown). However, it is reassuring to see that the variations of the EEG frequency profile over time appear as small perturbations around a largely invariant average power spectrum. More precisely, the appearance of GSW activity does not dramatically distort its shape, but rather induces frequency modulations of small amplitude. This is important, since this affords face validity to the qualitative prediction of our neural field model. Interestingly, there is a clear change in the normalized frequency power beginning at the onset of the second GSW crisis. This leads to a marked decrease in the EEG center frequency (the first GSW event seems to be associated with a similar but weaker effect). We will briefly comment on GSW-related frequency modulations in the discussion.

**Figure 6:**
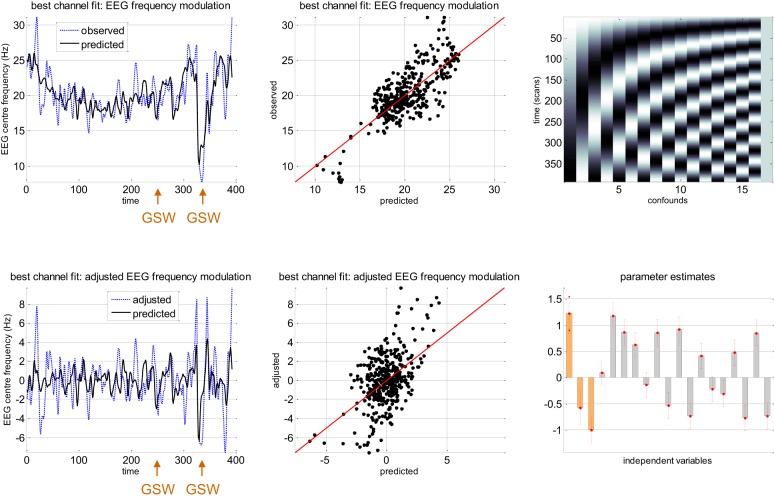
**Absence seizure analysis: EEG results (1).** This figure summarizes the results of the analysis on the EEG frequency modulation in a single subject (same patient as in Figure 5) at channel C4. **Upper-left:** Observed (blue dashed line) and predicted (black plain line) frequency modulation (y-axis) of the best EEG channel (C4) as a function of scanning time (x-axis). For this patient, the two GSW episodes are indicated by the orange arrows. **Upper-middle:** Observed (y-axis) versus predicted (x-axis) frequency modulation for EEG channel C4. **Upper-right:** Matrix of confounds **X**_0_ included in the analysis (slow drifts). **Lower-left:** Observed (blue dashed line) and predicted (black plain line) frequency modulation (y-axis) of the best EEG channel (C4) as a function of scanning time (x-axis), after adjustment for confounds. **Lower-middle**: Adjusted (y-axis) versus predicted (x-axis) frequency modulation for EEG channel C4. **Lower-right:** GLM parameter estimates, ± one standard deviation (orange: β, gray: β_0_).

Figure 6 summarizes the relationship between slow macroscopic sDCM states and the EEG frequency modulation of channel C4 for the same patients as in previous figures. Even though one can see that the center frequency $\bar{\omega }$ is very well fitted by the model (upper row of Figure 6: coefficient of determination: *R*^2^ = 0.59), one might think that most of the frequency modulation could be explained by confounds. For example, eyeballing the trajectory of the observed EEG center frequency reveals clear low frequency tends that are likely to be captured by slow drifts confounds. To disclose the specific contribution of neural state estimates, we have plotted the data and model prediction after removing the confounds (adjusted data). One can see that, when considering slow drifts confounds, sDCM neuronal estimates explain EEG frequency modulations of about 10 Hz. This is a similar order of magnitude than center frequency variations captured by slow drifts (cf. parameter estimates on lower-right panel of Figure 6). Note that the corresponding correlation coefficient is about 0.49 (adjusted *R*^2^ = 0.24), which indicates a reasonably good fit (above and beyond confounds).

**Figure 7:**
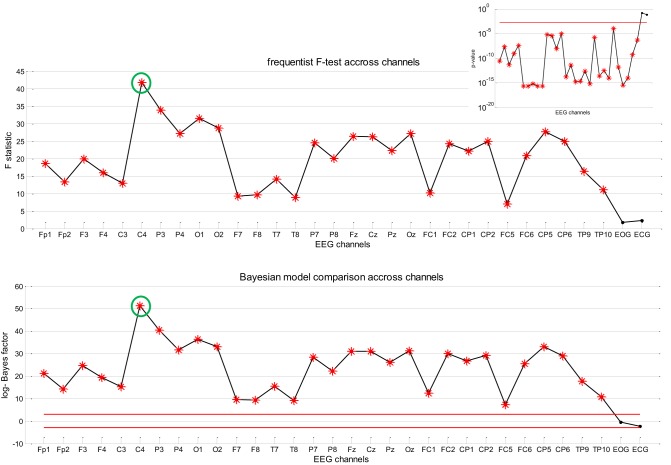
**Absence seizure analysis: EEG results (2).** This figure summarizes the results of the analysis on the EEG frequency modulation in a single subject (same patient as in Figures 5, 6) across channels. **Upper panel**: Value of the *F*-statistic (y-axis) when testing for the significance of the contribution of the sDCM neural states dynamics in the EEG frequency modulation, as a function of EEG channels (x-axis). Red stars indicate channels that pass the 5% false positive rate threshold (with Bonferroni correction of the multiple comparisons across channels). **Upper-right inset**: Associated *p*-value across EEG channels (the red line indicates the corrected 5% threshold). **Lower panel**: Log Bayes factor log *p*(*H*_1_|*y*) − log *p*(*H*_0_|*y*) showing evidence in favor of *H*_1_ versus *H*_0_ across EEG channels (see main text). Red lines indicate posterior probabilities of model *H*_1_ of 95% (upper line) and 5% (lower line), respectively.

Figure 7 summarizes the result of Bayesian model comparison, in terms of the log-Bayes factor $\text{LBF}=\log p(\bar{\omega }|H_{1})-\log p(\bar{\omega }|H_{0})$, across all EEG channels for the same patient as before. One can see that all EEG channels show strong evidence in favor of the full model (*H*_1_), whereas there is evidence against *H*_1_ only for EOG and ECG channels. This suggests that the neuronal fluctuations estimated by stochastic DCM predict the EEG frequency modulation beyond slow drifts confounds. For completeness, we also show the results of classical inference (*F*-test with significance level *p* = 0.05, full model *H*_1_ against the null *H*_0_). As this test was repeated independently for each of the 30 channels, we applied a Bonferroni correction for multiple comparisons across channels. One can see that the profile of *F*-statistics and log-Bayes factors (LBFs) across channels is very similar.

Recall that the results summarized in Figures 6, 7 are only shown to illustrate the analysis. In fact, it is important to note that they (1) depend upon the definition of the confounds (here slow drifts) and (2) do not capture inter-individual variability. The question of whether the accuracy with which the neural states inferred by sDCM are able to predict the frequency modulations of the EEG signal (above and beyond confounds) generalizes at the group level is addressed below.

This Bayesian model comparison for the single patient shown in Figure 7 was then repeated for all subjects, for both sets of confounds (namely: slow drifts and full confounds). First, we pooled evidence over EEG channels for each subjects. This was simply done by summing the log- model evidences over EEG channels (within-subjects fixed effect. The ensuing distribution of LBFs across subjects—shown in Table 3 below—indicates that most subjects show very strong evidence in favor of *H*_1_, irrespective of the particular set of confounds.

**Table 3:** **Log-Bayes factors (LBF) of *H*_1_ versus *H*_0_, for all subjects, and both sets of confounds**.

**Subject**	**LBF (*H*_1_ against slow drifts)**	**LBF (*H*_1_ against full confounds)**
1	670.9	198.6
2	204.3	103.9
3	51.4	-4.8
4	92.9	23.3
5	17.9	185.4
6	4.4	35.3
7	-1.8	65.0
8	1.2	28.6
9	101.4	37.2

*A positive LBF indicates evidence in favor of H_1_ (|LBF|> 3: significant effect at the subject-level, Kass and Raftery, 1995). Highlighted cells depict the two subjects that show relative evidence in favor of H_0_*.

To confirm this, we ran a random-effect analysis (Stephan et al., 2009) to assess the prevalence of *H*_1_ and *H*_0_ at the group level. Note that the two sets of confounds (“slow drifts” and “full confounds”) induce the four following models:

- *H*^(drifts)^_1_ : *H*_1_ with slow drifts
- *H*^(full)^_1_ : *H*_1_ with full confounds
- *H*^(drifts)^_0_ : *H*_0_ with slow drifts
- *H*^(full)^_0_ : *H*_0_ with full confounds.

**Figure 8:**
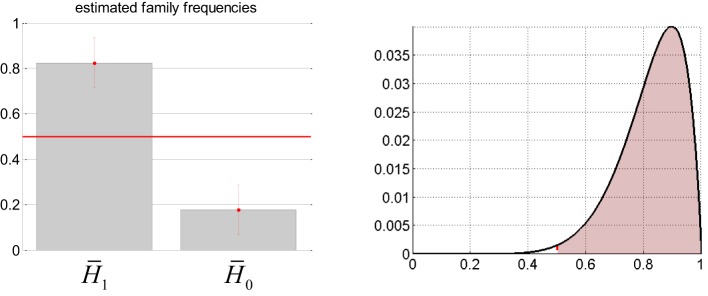
**Absence seizure analysis: EEG results (3).** This figure summarizes the results of the analysis on the EEG frequency modulation at the group level (across subjects). **Left**: Posterior estimates of the expected frequency of families ${\bar{H}}_{0}$ and within the population (± one posterior standard deviation). **Right**: Posterior density (y-axis) over the expected frequency (x-axis) of family ${\bar{H}}_{1}$ within the population. The red shaded area indicates the mass of probability that is beyond 50% prevalence, yielding an exceedance probability of about 99%.

The comparison of interest is in terms of whether or not adding the sDCM slow neural states adds anything to the prediction of the EEG center frequency, above and beyond confounds. Thus, we applied group-level family inference (Penny et al., 2010) to evaluate the evidence in favor or against *H*_1_, irrespective of the definition of the confounds. We have thus partitioned the set of four models above into two families ${\bar{H}}_{1}$ and ${\bar{H}}_{0}$ that gather both sorts of confounds, as follows: ${\bar{H}}_{1}=\left\{H_{1}^{\left(\text{drifts}\right)},H_{1}^{\left(\text{full}\right)}\right\}$ and ${\bar{H}}_{0}=\left\{H_{0}^{\left(\text{drifts}\right)},H_{0}^{\left(\text{full}\right)}\right\}$. Deriving the relative evidence of ${\bar{H}}_{1}$ against ${\bar{H}}_{0}$ effectively marginalizes over the different confounds. First of all, we checked that there was a random effect at all, i.e.,: a difference between the respective prevalence of all models within the population. We found that the posterior probability of all models being equally frequent was *p* = 0.03. This Bayesian omnibus test indicates that the distribution of Bayes factors shown in Table 3 is unlikely to be due to chance. Figure 8 summarizes the group-level family inference results. One can see that the estimated prevalence of family ${\bar{H}}_{1}$ is about 0.82. In addition, the exceedance probability of ${\bar{H}}_{1}$ (versus ${\bar{H}}_{0}$) was 0.991. In other words, we can be 99% confident that sDCM estimates of neuronal fluctuations predict concurrent EEG frequency modulation above and beyond physiological confounds (in more than half the population).

For completeness, we have also performed bayesian group-level model comparison, conditional on the definition of confounds. The results match those of the family-inference analysis above. In brief, the exceedance probability of *H*^(drifts)^_1_ (versus *H*^(drifts)^_0_) was 0.997, and the exceedance probability of *H*^(full)^_1_ (versus *H*^(full)^_0_) was 0.990. However, the bayesian omnibus test for these analyses was not as conclusive (slow drifts: *p* = 0.11, full confounds: *p* = 0.14). Taken together, this means that there is statistical evidence in favor of *H*_1_ (versus *H*_0_), irrespective of the definition of the confounds. This concludes our quantitative assessment of the predictive validity of sDCM for fMRI with regard to concurrently obtained EEG measures.

## Discussion

Introducing random neural fluctuations in DCM for fMRI was originally motivated by the need to estimate hidden states in the absence of experimental perturbations. In this note, we have assessed the predictive validity of sDCM estimates of slow macroscopic neural states with respect to EEG frequency modulation. We have revisited the heuristic hemodynamic correlate of the EEG proposed in Kilner et al. (2005), using a neural field formulation to show how slow modes of local electrophysiological activity (which we assume drive changes in the fMRI signal) enslave the frequency spectrum of fast modes that contribute to the EEG signal. From this theoretical perspective, we tested whether slow neural estimates obtained by sDCM could explain concurrent EEG frequency modulation above and beyond physiological confounds.

To our knowledge, this work is the first attempt to provide empirical evidence that neural fluctuations inferred using stochastic DCM from fMRI time series have an electrophysiological underpinning. From an experimental perspective, this endorses the use of sDCM for studying spontaneous fluctuations that are beyond experimental control. Examples of this include—but are not limited to—multistable perception (Hesselmann et al., 2010) and epileptic events (e.g., absence seizures, cf. Vaudano et al., 2009). In this context, the added-value of sDCM is that it allows for comparing quantitative hypotheses about the propagation and/or the emergence of these phenomena within brain networks.

The acute reader might question the relevance of the modeling part of our paper, in the sense that quantitative predictions of the model were not used in the subsequent data analysis. However, we believe this model may contribute to the debate regarding the hemodynamic correlate of the EEG. First, it bears a number of interesting differences, with respect to the heuristic model of Kilner et al. (2005):

- Here, the main prediction, namely that fMRI activation is associated with a frequency modulation of the EEG signal, only relies on the assumption that BOLD changes are mostly driven by the fundamental (slow and smooth) eigenmode of the membrane potential field. This differential sensitivity of EEG and fMRI with respect to higher-order canonical eigenmodes bypasses most modeling assumptions of the heuristic model.
- Here, the relationship between activation and the EEG center frequency is not monotonic. More precisely, the center frequency decreases as one approaches the action potential threshold, either from hyperpolarized states (activation) of from depolarized states (de-activation). In brief, this form of critical slowing is qualitatively equivalent to the heuristic model, above the action potential threshold.

Second, we believe that the above qualitative theoretical prediction is very robust to model assumptions. First, we checked that the effect persisted in the context of our model, when changing the parameters that control the biophysical properties of the neural field (results not shown). Second, the effect is a byproduct of local propagation of activity over the field. In brief, smooth local patterns emerge in densely connected brain regions, the—slow—dynamics of which are shaped by effective connectivity between remote regions. Each local eigenmode is then expected to control the acceleration or slowing down of the response of its respective brain region. In contradistinction to e.g., quantitative predictions about frequency power spectra, we anticipate this qualitative prediction to hold, irrespective of models' specificities.

Let us now consider the (modest) theoretical contribution of this work in relation to the separation of time scales. We believe that—if properly extended—established biophysical models of fast electrophysiological responses may be a good starting point for understanding slow dynamical modes at macroscopic spatial scales that, presumably, drive fMRI responses. The neural field treatment we have developed in this note is a first step toward a mechanistic understanding of the macroscopic dynamics that emerge at the time scale of seconds or minutes. However, this model is limited in many ways. First, we did not account for extrinsic connectivity between regions (coupled neural fields). This is known to strongly impact on the stability of spatiotemporal brain dynamics (Knock et al., 2009), and will thus be critical for predicting the dynamical repertoire of slow macroscopic modes of activity (cf. point above). For example, Bojak et al. (2011) show that increasing the long-range coupling between remote regions strongly modulates the frequency response of the target region, in terms of the relative power in low- and high- frequency bands. Second, we did not separate the respective contributions of excitatory and inhibitory subpopulations (Pinotsis et al., 2012). Of particular importance here is the fact that inhibito-excitatory networks can possess stable limit cycles (Seung et al., 1995). This means that local neural fields can behave as oscillators or resonators. This relates to the work of Cabral et al. (2011) who examined the role of local network oscillations in the emergence of slow temporal synchrony between remote brain areas. The critical thing here is that the time it takes for one resonator to influence another resonator can be much slower than the local resonance frequencies. In addition, owing to the autonomous nature of local dynamics, the effect of between-regions coupling might be better understood in terms of changes in the resonance frequency of the target region. Taken together, these considerations suggest that challenging theoretical work still needs to be undertaken to establish the dynamical repertoire of coupled slow macroscopic modes of brain activity.

We take the fact that the sDCM neural states contribution was significant for all EEG channels (above and beyond slow drifts) as a compelling validation of the conditional estimates of neuronal activity from sDCM. Importantly, this also provides some empirical evidence in favor of the fMRI to EEG mapping itself. This supports the results of Rosa et al. (2010b), which is, to our knowledge, the only experimental study that tested predictions of the heuristic model of Kilner et al. (2005). In this study, the authors experimentally controlled the frequency content of the EEG signal, and showed that the BOLD response in a group subjects was better explained by frequency modulation than by amplitude modulation of the EEG. More specifically, Rosa et al. (2010b) showed that to correctly model BOLD variations it was important to use a normalized frequency spectrum, as in Equation (10). Here, we approach this issue from the other side and use fMRI to predict the EEG frequency spectrum. Note that this is only an indirect validation of the neurovascular coupling model, as we did not compare it with other quantitative scenarios. Among these is the idea that BOLD changes correlate with amplitude modulations within frequency bands of interest in the EEG spectrum. Typically, the BOLD signal is considered a filtered version of the EEG alpha power (see e.g., De Munck et al., 2008). More generally, this idea has been applied to each and every EEG frequency band with relative success [see Laufs et al. (2008) and references therein]. As an illustration, Lachaux et al. (2007) have found that EEG gamma band modulations co-localize with BOLD variations. However, although power fluctuations of different EEG frequency bands are known to be mutually highly correlated, multi frequency models are usually not used in such studies (De Munck et al., 2009). In addition to compromising the specificity of the results, this prevents a direct comparison with our and related work (cf. Rosa et al., 2010b).

Another limitation of our data analysis is that we neglected the unavoidable non-linearity implicit in the relationship $\bar{\omega }=\bar{\omega }\left(z_{1}^{\left(0\right)}\right)$ (cf. Equations 9–12 and Figure 2) when testing for the contribution of slow neural states to modulations of the EEG center frequency. This means we may have not taken full advantage of our neural field formulation. However, this does not detract from our quantitative assessment of the predictive validity of sDCM for fMRI data.

When examining the GSW-related EEG frequency modulations, we noted that the frequency spectrum did not change markedly during GSW activity. This might seem surprising, if one thinks of GSW as sudden bursts of activity within the network, associated with clearly recognizable 3 Hz transient waves of EEG activity (Destexhe and Sejnowski, 2001). Closer inspection of the EEG frequency profile confirmed the appearance of a peak at 3 Hz during GSW-activity (not shown). However, many other (faster) processes actually contribute to the overall frequency spectrum, which typically drags the center frequency toward 20 Hz. This is the main reason why we had to include as many confounding factors as possible, when assessing the specific contribution of the sDCM predictors. Importantly, physiological confounds (cardiac activity, head movements, etc.) span most of the slow frequencies in the EEG signal, which potentially masks interesting slow neural processes. This means that slow frequency EEG signals with a neural origin are confounded with physiological noise. Note that there are notorious exceptions to this detectability issue, for example readiness potentials (Jahanshahi and Hallett, 2003) that are thought to reflect the preparation of motor activity and have a typical time scale of a few seconds. Nevertheless, our results indicate that it may be possible to detect the contribution of slow neural processes to the scalp EEG signal, through their indirect effect on the frequency modulation of fast modes.

In conclusion, we have provided empirical evidence supporting the predictive validity of sDCM for fMRI data. In future work, we will examine the empirical and theoretical relationships that exist between slow and fast dynamical consequences of effective connectivity as can be accessed via fMRI and EEG, respectively.

## Conflict of interest statement

The authors declare that the research was conducted in the absence of any commercial or financial relationships that could be construed as a potential conflict of interest.

## Appendix 1: approximating the local neural fields with a spatial eigenmodes expansion

Let ∇^2^ = ∂^2^/∂*x*^2^ +∂^2^/∂*y*^2^ be the 2D-Laplacian operator defined on a bounded (square) 2D domain of size ‖D‖, where {*x*, *y*} = **r** are the two cartesian coordinates on the domain. Recall that eigenvectors *w*^(*k, l*)^ (**r**) of ∇^2^ are such that:

(A1) $$\begin{array}{l} w^{\left(k,\;l\right)}(r)=e^{i\frac{\pi }{\left\|D\right\|}(kx\;+\;ly)}\\ \;=w^{\left(k\right)}(x)w^{\left(l\right)}(y)\\ \;={\left[w^{\left(1\right)}(x)\right]}^{k}{\left[w^{\left(1\right)}(y)\right]}^{l} \end{array}$$

where we have used index pairs (*k, l*) for spatial frequencies along the x- and y-axis, respectively. The associated eigenvalues are given by: $\lambda_{k,\;l}=-\left(k^{2}+l^{2}\right)\pi^{2}/\|D{\|}^{2}$. By convention, *w*^(*k*)^ refer to the eigenfunctions of the 1D-Laplacian, and its complex conjugate will be denoted as *w*^(−*k*)^. Now, projecting Equation (5) onto the (complex conjugate) spatial eigenmodes yields the eigendynamics of the neural field (Daunizeau et al., 2009a):

(A2) $$\begin{array}{l} \tau^{2}{\ddot{z}}_{1}^{\left(k,\;l\right)}(t)=-2\tau {\dot{z}}_{1}^{\left(k,\;l\right)}(t)-z_{1}^{\left(k,\;l\right)}(t)+\tau \gamma z_{2}^{\left(k,\;l\right)}(t)\\ \;{\dot{z}}_{2}^{\left(k,\;l\right)}(t)=\frac{\bar{\nu }}{2\sigma }\left(\left(\sigma^{2}\lambda_{kl}-1\right)z_{2}^{\left(k,\;l\right)}(t)+G^{0}\underset{\varsigma_{k,\;l}(t)}{\underset{︸}{\int_{D}w^{\left(-k,\;-l\right)}(r)S\left(\eta (r,t)\right)dr}}\right). \end{array}$$

where the projections ς_*k*, *l*_ (*t*) of the firing rate form a complete basis function set.

Together with a truncated eigenmode expansion, Equation (A2) can be used to approximate the solution to Equation (5) of the main text. Such a truncation basically acts as a low-pass filter, neglecting the transient effects of higher spatial frequency modes. This can be motivated by noting that (1) the eigenmodes *w*^(k, l)^ (r) of the Laplacian operator are of increasing spatial frequency as the order (k, l) increases and (2) the associated eigenvalues λ_*kl*_ are negative (except for λ_00_ = 0) with increasing magnitude. This implies that the dynamics of high-order spatial modes will be strongly damped and thus quickly disappear in the absence of external forcing.

We now focus on deriving an approximation to the projections ς_*k*, *l*_ (*t*) of the firing rate.

First, let us Taylor-expand the sigmoidal mapping of PSP activity around zero:

(A3) $$S(\eta (r,\;t))=\sum_{m\;=\;0}^{\infty }\frac{1}{m!}S_{0}^{\left[m\right]}\eta^{m}(r,\;t),$$

where $S_{0}^{\left[m\right]}={\frac{\partial^{m}S}{\partial x^{m}}|}_{0}$ is the *m*th derivative of the sigmoidal mapping evaluated at η = 0 and η^*m*^ (**r**, *t*) is the *m*th power of mean PSP η(**r**, *t*). Assuming that Equation (A1) holds for the eigenvectors of the Laplacian operator defined onto the cortical domain *D*, Equation (6) becomes:

(A4) $$\begin{array}{l} \;\eta (r,\;t)=\sum_{k}\sum_{l}z_{1}^{\left(k,\;l\right)}(t)w^{\left(k,\;1\right)}(r)\\ \;=\sum_{k}{\tilde{z}}_{1}^{\left(k\right)}(t,\;y){\left[w^{\left(1\right)}(x)\right]}^{k}\\ {\tilde{z}}_{1}^{\left(k\right)}(t,\;y)\equiv \sum_{l}z_{1}^{\left(k,\;l\right)}(t){\left[w^{\left(1\right)}(y)\right]}^{l} \end{array}$$

where ${\tilde{z}}_{1}^{\left(k\right)}(t,\;y)$ is a dummy eigenmode dynamics, that is still a function of space (more precisely, of the second coordinate *y*). Inserting Equation (A4) into (A3) yields the following power series of power series in *w*^(1)^:

(A5) $$\begin{array}{l} S(\eta (r,\;t))=\sum_{m\;=\;0}^{\infty }\frac{1}{m!}S_{0}^{\left[m\right]}{\left(\sum_{k}{\tilde{z}}_{1}^{k}(t,\;y){\left[w^{\left(1\right)}(x)\right]}^{k}\right)}^{m}\\ \;=\sum_{m\;=\;0}^{\infty }\frac{1}{m!}S_{0}^{\left[m\right]}\sum_{k}A_{k}(t,y,m)w^{\left(k\right)}(x) \end{array}$$

where the coefficients *A*_*k*_ (*t, y, m*) obey the following recursive equation (Von Holdt, 1965):

(A6) $$\left\{ \begin{array}{l} A_{k}(t,y,m)=\frac{1}{k{\tilde{z}}_{1}^{\left(0\right)}(t)}\sum_{i\;=\;1}^{k}\left((m+1)i-k){\tilde{z}}_{1}^{\left(i\right)}(t)A_{k\;-\;i}(t,y,m\right)\\ A_{0}(t,y,m)={\left[{\tilde{z}}_{1}^{\left(0\right)}(t)\right]}^{m} \end{array}\right.$$

The contribution ϒ_*k*_(*t, y*) of the *k*th spatial mode to the expansion in Equation (A5) is given by:

(A7) $$\begin{array}{l} S(\eta (r,t))=\sum_{k}{ϒ}_{k}(t,y)w^{\left(k\right)}(x)\\ \;{ϒ}_{k}(t,y)=\sum_{m\;=\;0}^{\infty }\frac{1}{m!}S_{0}{}^{\left[m\right]}A_{k}(t,y,m) \end{array}$$

These can be analytically derived from the recursive relationship given in Equation (A6). They are given in Table A1 bellow, up to order *k* = 3.

**Table A1:** **This table gives both the coefficients *A*_*k*_ (*t*, *y*, *m*) (left) and the contribution of the *k*th spatial mode to the expansion in Equation (A4), as given by ϒ_*k*_(*t*, *y*) (right) up to *k* = 3**.

	***A*_*k*_(*t, y, m*)**	**ϒ_*k*_(*t*, *y*)**
*k* = 0	${\left[{\tilde{z}}_{1}^{\left(0\right)}\right]}^{m}$	$S\left({\tilde{z}}_{1}^{\left(0\right)}\right)$
*k* = 1	$\frac{1}{1!}m{\tilde{z}}_{1}^{\left(1\right)}{\left[{\tilde{z}}_{1}^{\left(0\right)}\right]}^{m\;-\;1}$	$\frac{1}{1!}{\tilde{z}}_{1}^{\left(1\right)}S^{\prime }\left({\tilde{z}}_{1}^{\left(0\right)}\right)$
*k* = 2	$m{\tilde{z}}_{1}^{\left(2\right)}{\left[{\tilde{z}}_{1}^{\left(0\right)}\right]}^{m\;-\;1}+\frac{m!}{2!(m-2)!}{\left[{\tilde{z}}_{1}^{\left(1\right)}\right]}^{2}{\left[{\tilde{z}}_{1}^{\left(0\right)}\right]}^{m\;-\;2}$	${\tilde{z}}_{1}^{\left(2\right)}S^{\prime }\left({\tilde{z}}_{1}^{\left(0\right)}\right)+\frac{1}{2!}{\left[{\tilde{z}}_{1}^{\left(1\right)}\right]}^{2}S^{\prime \prime }\left({\tilde{z}}_{1}^{\left(0\right)}\right)$
*k* = 3	$\begin{array}{l} m{\tilde{z}}_{1}^{\left(3\right)}{\left[{\tilde{z}}_{1}^{\left(0\right)}\right]}^{m\;-\;1}+\frac{m!}{(m-2)!}{\tilde{z}}_{1}^{\left(1\right)}{\tilde{z}}_{1}^{\left(2\right)}{\left[{\tilde{z}}_{1}^{\left(0\right)}\right]}^{m\;-\;2}+\frac{m!}{3!(m-3)!}{\left[{\tilde{z}}_{1}^{\left(1\right)}\right]}^{3}{\left[{\tilde{z}}_{1}^{\left(0\right)}\right]}^{m\;-\;3} \end{array}$	$\begin{array}{l} {\tilde{z}}_{1}^{\left(3\right)}S^{\prime }\left({\tilde{z}}_{1}^{\left(0\right)}\right)+{\tilde{z}}_{1}^{\left(1\right)}{\tilde{z}}_{1}^{\left(2\right)}S^{\prime \prime }\left({\tilde{z}}_{1}^{\left(0\right)}\right)+\frac{1}{3!}{\left[{\tilde{z}}_{1}^{\left(1\right)}\right]}^{3}S^{\left[3\right]}\left({\tilde{z}}_{1}^{\left(0\right)}\right) \end{array}$

It can be seen form Table A1 that, except for the fundamental mode, the spatial mode contribution ϒ_*k*_ is of the form:

(A8) $$\begin{array}{l} {ϒ}_{k}\propto \sum_{j\;=\;1}^{k}\text{O}\left({\left[{\tilde{z}}_{1}\right]}^{j}\right)S^{\left[j\right]}\left({\tilde{z}}_{1}^{\left(0\right)}\right)\\ \;=\sum_{j\;=\;1}^{k}\text{O}\left({\left[{\tilde{z}}_{1}\right]}^{j}\right)\rho^{j}S\left({\tilde{z}}_{1}^{\left(0\right)}\right)\prod_{m\;=\;1}^{j}\left(1-mS\left({\tilde{z}}_{1}^{\left(0\right)}\right)\right),\\ \;\approx \sum_{j\;=\;1}^{k}\text{O}\left({\left[{\tilde{z}}_{1}\right]}^{2j\;+\;1}\right) \end{array}$$

where O(*x*^j^) is a polynomial of order *j* in *x* and ρ is the sigmoidal slope. The second line in Equation (A8) follows from the following property of the sigmoid function: *S*^[*j*]^ (*x*) = ρ^j^ *S*(*x*) ∏^j^_*m* = 1_(1 − *mS*(*x*)) and the last line would follow from a first-order Taylor expansion of the sigmoidal mapping. This means that the lower order term in each spatial mode contribution ϒ_*k*_ (*t, y*) is already a polynomial of order three in the dummy eigenmodes dynamics ${\tilde{z}}_{1}^{\left(k\right)}(t,y)$. We will therefore truncate the contribution to its first term, i.e.,: ${ϒ}_{0}(t,y)=S\left({\tilde{z}}_{1}^{\left(0\right)}(t,y)\right)$ for *k* = 0, and ${ϒ}_{k}(t,y)\approx {\tilde{z}}_{1}^{\left(k\right)}(t,y)S^{\prime }\left({\tilde{z}}_{1}^{\left(0\right)}(t,y)\right)$ for *k*>0. This yields the following approximation to the firing rate field:

(A9) $$S(\eta (r,t))\approx S\left({\tilde{z}}_{1}^{\left(0\right)}(t,y)\right)w^{\left(0\right)}(x)+\sum_{k\;\geq \;0}\rho S^{\prime }\left({\tilde{z}}_{1}^{\left(0\right)}(t,y)\right){\tilde{z}}_{1}^{\left(k\right)}(t,y)w^{\left(k\right)}(x)$$

Equation (A9) can now be used to approximate the integral with respect to *x* in Equation (A2):

(A10) $$\begin{array}{l} \varsigma_{k,\;l}(t)=\int w^{\left(-l\right)}(y)\left[\int w^{\left(-k\right)}(x)S(\eta (r,t))dx\right]\text{​}dy\\ \;\approx \delta_{k}\int w^{\left(-l\right)}(y)S\left({\tilde{z}}_{1}^{\left(0\right)}(t,y)\right)dy+(1-\delta_{k})\rho \int w^{\left(-l\right)}(y)S^{\prime }({\tilde{z}}_{1}^{\left(0\right)}(t,y)){\tilde{z}}_{1}^{\left(k\right)}(t,y)dy\\ \;=\delta_{k}\int w^{\left(-l\right)}(y)S\left({\tilde{z}}_{1}^{\left(0\right)}(t,y)\right)dy+(1-\delta_{k})\rho \sum_{l^{\prime }}z_{1}^{k,\;l}(t)\int w^{\left(l^{\prime }\;-\;l\right)}(y)S^{\prime }\left({\tilde{z}}_{1}^{\left(0\right)}(t,y)\right)\text{​}dy \end{array}$$

where δ_*k*_ is a Dirac delta, which is zero except when *k* = 0.

Similarly, the sigmoidal terms in the integrands of Equation (A10) can be approximated using power series of power series, as follows:

(A11) $$\begin{array}{l} \;S\left({\tilde{z}}_{1}^{\left(0\right)}(t,y)\right)\approx S\left(z_{1}^{\left(0,\;0\right)}(t)\right)w^{\left(0\right)}(y)+\sum_{l\;\geq \;0}\rho S^{\prime }\left(z_{1}^{\left(0,\;0\right)}(t)\right)z_{1}^{\left(0,\;l\right)}(t)w^{\left(l\right)}(y)\\ S^{\prime }\left({\tilde{z}}_{1}^{\left(0\right)}(t,y)\right)\approx S^{\prime }\left(z_{1}^{\left(0,\;0\right)}(t)\right)w^{\left(0\right)}(y)+\sum_{l\;\geq \;0}\rho S^{\prime \prime }\left(z_{1}^{\left(0,\;0\right)}(t)\right)z_{1}^{\left(0,\;l\right)}(t)w^{\left(l\right)}(y) \end{array}$$

Again, inserting Equation (A11) into (A10), and ignoring high-order terms^1^ yields:

(A12) $$\varsigma_{k,\;l}(t)=\delta_{k}\delta_{l}S\left(z_{1}^{\left(0,\;0\right)}(t)\right)+\left(1-\delta_{k}\delta_{l}\right)\rho S^{\prime }\left(z_{1}^{\left(0,\;0\right)}(t)\right)z_{1}^{\left(k,\;l\right)}(t)$$

Inserting Equation (A12) in (A2) yields:

(A13) $${\dot{z}}_{2}^{\left(k,l\right)}(t)=\left\{ \begin{array}{l} \frac{\bar{\nu }}{2\sigma }\left(G^{0}S\left(z_{1}^{\left(0,\;0\right)}(t)\right)-z_{2}^{\left(0,\;0\right)}(t)\right)\text{ if}\;k=l=0\\ \frac{\bar{\nu }}{2\sigma }\left(\left(\sigma^{2}\lambda_{kl}-1\right)z_{2}^{\left(k,\;l\right)}(t)+\rho G^{0}S^{\prime }\left(z_{1}^{\left(0,\;0\right)}(t)\right)z_{1}^{\left(k,\;l\right)}(t)\right)\text{ otherwise} \end{array}\right.$$

The second line of Equation (A13) is Equation (8) of the main text, having re-ordered the eigenmodes index pairs (*k, l*) → *k*, for the sake of notational simplicity.

The critical properties of this derivation can be summarized as follows:

First, the fundamental eigenmode response will be slower than higher-order eigenmodes (because of smaller dampening terms). Second, the response of fast eigenmodes is enslaved by the slow eigenmode. Third, the fast eigenmodes do not feedback onto the slow eigenmodes. Taken together, we can think of the neural field as a feedforward system: inputs to the field activate the slow eigenmode *z*^(0, 0)^_1_ (*t*), which controls the fast modes *z*^(k, l)^_1_ (*t*). This is further discussed in the main text of this document.

## Appendix 2: deriving the frequency spectrum of eigenmodes

Recall that the eigenmode dynamics obey the following ODE (cf. Equation 8 in the main text):

(A14) $$\begin{array}{l} {\dot{z}}^{\left(k\right)}=Az^{\left(k\right)}+\nu \\ \;A=\left[\begin{array}{ccc} 0 & 0 & 1\\ c\left(z_{1}^{\left(0\right)}\right) & -\frac{\bar{\nu }}{2\sigma }\left(1-\sigma^{2}\lambda_{k}\right) & 0\\ -\frac{1}{\tau^{2}} & \frac{\gamma }{\tau } & -\frac{2}{\tau } \end{array}\right] \end{array}$$

where ν = O(*z*^2^) are high-order error terms that act as a perturbation to the deterministic evolution of eigenmodes. As a simplifying assumption, we consider that ν behaves as i.i.d. white noise, the Fourier transform *F*[*z*^(*k*)^] of Equation (A8) writes:

(A15) $$\begin{array}{l} F\left[z^{\left(k\right)}\right]={\left(2i\pi \omega I_{3}-A\right)}^{-1}F[\nu ]\\ \;\propto {\left(2i\pi \omega I_{3}-A\right)}^{-1}1_{3} \end{array}$$

where **1**_3_ is a 3 × 1 vector of ones, and **I**_3_ is the 3 × 3 identity matrix.

Note that the inverse translated Jacobian writes:

(A16) $$\begin{array}{l} {\left(2i\pi \omega I_{3}-A\right)}^{-1}=\frac{1}{\left|2i\pi \omega I_{3}-A\right|}\left[\begin{array}{ccc} \xi_{k}(\omega )\left(i\omega +\frac{2}{\tau }\right) & \frac{\gamma }{\tau } & \xi_{k}(\omega )\\ c\left(z_{1}^{\left(0\right)}\right)\left(i\omega +\frac{2}{\tau }\right) & i\omega \left(i\omega +\frac{2}{\tau }\right)+\frac{1}{\tau^{2}} & c\left(z_{1}^{\left(0\right)}\right)\\ \frac{\gamma }{\tau }c\left(z_{1}^{\left(0\right)}\right)-\xi_{k}(\omega )\frac{1}{\tau^{2}} & i\omega \frac{\gamma }{\tau } & i\omega \xi_{k}(\omega ) \end{array}\right]\\ \;\xi_{k}(\omega )=i\omega +\frac{\bar{\nu }}{2\sigma }\left(1-\sigma^{2}\lambda_{k}\right) \end{array}$$

where ξ_*k*_ (ω) is the only term that depends upon the mode order *k*, and the determinant of the translated Jacobian is given by:

(A17) $$\begin{array}{l} \left|2i\pi \omega I_{3}-A\right|=i\omega \xi_{k}(\omega )\left(i\omega +\frac{2}{\tau }\right)-\frac{\gamma }{\tau }c\left(z_{1}^{\left(0\right)}\right)+\xi_{k}(\omega )\frac{1}{\tau^{2}}\\ \;=\xi_{k}(\omega )\left(i\omega \left(i\omega +\frac{2}{\tau }\right)+\frac{1}{\tau^{2}}\right)-\frac{\gamma }{\tau }c\left(z_{1}^{\left(0\right)}\right) \end{array}$$

where ξ_*k*_ (ω) is given in Equation (A12) (with appropriate eigenmodes index reordering (*k, l*) → *k*). The explicit Fourier transform of eigenmode dynamics now derives from inserting Equation (A16) into (A15):

(A18) $$F\text{​}\left[z^{\left(k\right)}\right]\propto \frac{1}{\left|2i\pi \omega I_{3}-A\right|}\left[\begin{array}{c} \xi_{k}(\omega )\left(i\omega +\frac{2}{\tau }+1\right)+\frac{\gamma }{\tau }\\ \left(i\omega +\frac{2}{\tau }\right)\left(c\left(z_{1}^{\left(0\right)}\right)+i\omega \right)+\frac{1}{\tau^{2}}+c\left(z_{1}^{\left(0\right)}\right)\\ \xi_{k}(\omega )\left(i\omega -\frac{1}{\tau^{2}}\right)+\frac{\gamma }{\tau }\left(i\omega +c\left(z_{1}^{\left(0\right)}\right)\right) \end{array}\right]$$

Now, we are interested in the total frequency power of EEG signal sampled at some position *r*' on the scalp that is induced by mean membrane depolarization:

(A19) $$\begin{array}{l} \text{P}(\omega ,r^{\prime })\equiv {\left|\int_{D}L(r,r^{\prime })F[\eta (r,t)]dr\right|}^{2}\\ \;={\left|\sum_{k}\alpha^{\left(k\right)}(r^{\prime })F\left[z_{1}^{\left(k\right)}\right]\right|}^{2}\\ \alpha^{\left(k\right)}(r^{\prime })=\int_{D}L(r,r^{\prime })w^{\left(k\right)}(r)dr \end{array}$$

where the second line derives from the eigendecomposition of the neural field (cf. Equation 6), *L*(**r, r**′) is a spatial convolution operator that describes the quasi-static volume propagation of electromagnetic fields through head tissues, and α^(*k*)^ (r′) captures the contribution of the *k*th eigenmode at position r′ on the scalp. It is known that the geometrical properties of electrical conductivity within head tissues eventually shape the EEG susceptibility to different cortical spatial scales. For the sake of simplicity, we will assume the skull acts as a perfect low-pass filter (see e.g., Srinivasan et al., 1998), which induces a cut-off order *k*_*c*_, i.e.,:

(A20) $$\alpha^{\left(k\right)}(r^{\prime })=\{\begin{array}{cc} \begin{array}{l} \alpha^{\left(0\right)}(r^{\prime })\\ 0 \end{array} & \begin{array}{l} \text{if }k\leq k_{c}\\ \text{if }k>k_{c} \end{array} \end{array}$$

Equation (A20) basically neglects the continuous loss of sensitivity as one increases the spatial frequency of (cortical) membrane depolarization fields. Inserting Equation (A20) into (A19) yields Equation (9) of the main text, i.e.,:

(A21) $$\begin{array}{l} \text{P}\left(\omega ,r^{\prime }\right)=\alpha^{\left(0\right)}{\left(r^{\prime }\right)}^{2}{\left|\sum_{k\;\leq \;k_{c}}F\left[z_{1}^{\left(k\right)}\right]\right|}^{2}\\ \;=\alpha^{\left(0\right)}{\left(r^{\prime }\right)}^{2}{\left|\sum_{k\;\leq \;k_{c}}\frac{\xi_{k}(\omega )\left(i\omega +\frac{2}{\tau }+1\right)+\frac{\gamma }{\tau }}{\xi_{k}(\omega )\left(i\omega \left(i\omega +\frac{2}{\tau }\right)+{\frac{1}{\tau }}^{2}\right)-\frac{\gamma }{\tau }c\left(z_{1}^{\left(0\right)}\right)}\right|}^{2} \end{array}$$

where α^(0)^ (**r**′) is a scaling factor that will be different for different EEG sensors.

Equation (A21) is a valid approximation of the EEG power spectrum at position **r**′ on the scalp generated by one local brain region. The generalization to a network of remote brain regions requires the specification of the relative contribution of each region, which depends on their relative distance with EEG scalp sensors. In principle, this can be derived from knowledge of the forward operator *L*(**r, r**′) (see e.g., De Munck, 1988). This is beyond the scope of the present study.

Lastly, note that the numerical values of the Laplacian's eigenvalues are approximated from the 2D-Euclidean case, as follows: $\lambda_{k,\;l}=-\left(k^{2}+l^{2}\right)\pi^{2}/\|D{\|}^{2}$. The effective frequency cut-off *k*_*c*_ is such that *k*^2^ + *l*^2^ ≤ *k*^2^_*c*_. In practice, we use *k*^2^_*c*_ = 16 and ‖D‖=1cm.
